# Mitochondria exert age-divergent effects on recovery from spinal cord injury

**DOI:** 10.1016/j.expneurol.2021.113597

**Published:** 2021-01-07

**Authors:** Andrew N. Stewart, Katelyn E. McFarlane, Hemendra J. Vekaria, William M. Bailey, Stacey A. Slone, Lauren A. Tranthem, Bei Zhang, Samir P. Patel, Patrick G. Sullivan, John C. Gensel

**Affiliations:** a Department of Physiology, College of Arts and Sciences, University of Kentucky, Lexington, KY 40536, USA; b Department of Neuroscience, College of Arts and Sciences, University of Kentucky, Lexington, KY 40536, USA; c Spinal Cord and Brain Injury Research Center, University of College of Medicine, College of Arts and Sciences, University of Kentucky, Lexington, KY 40536, USA; d Department of Statistics, College of Arts and Sciences, University of Kentucky, Lexington, KY 40536, USA; e Brain and Mind Research Institute Weill Cornell, Weill Cornell Medicine, New York, NY, 10021, USA

**Keywords:** Metabolism, Secondary injury, Bioenergetics, Mitochondrial Uncouplers, Mitochondrial oxidative damage, Neuroprotection

## Abstract

The extent that age-dependent mitochondrial dysfunction drives neurodegeneration is not well understood. This study tested the hypothesis that mitochondria contribute to spinal cord injury (SCI)-induced neurodegeneration in an age-dependent manner by using 2,4-dinitrophenol (DNP) to uncouple electron transport, thereby increasing cellular respiration and reducing reactive oxygen species (ROS) production. We directly compared the effects of graded DNP doses in 4- and 14-month-old (MO) SCI-mice and found DNP to have increased efficacy in mitochondria isolated from 14-MO animals. *In vivo*, all DNP doses significantly exacerbated 4-MO SCI neurodegeneration coincident with worsened recovery. In contrast, low DNP doses (1.0-mg/kg/day) improved tissue sparing, reduced ROS-associated 3-nitrotyrosine (3-NT) accumulation, and improved anatomical and functional recovery in 14-MO SCI-mice. By directly comparing the effects of DNP between ages we demonstrate that mitochondrial contributions to neurodegeneration diverge with age after SCI. Collectively, our data indicate an essential role of mitochondria in age-associated neurodegeneration.

## 1. Introduction

Metabolic processes shift throughout a lifespan as an inevitable consequence of aging (a thorough review of how aging affects brain metabolism can be found in Mattson and Arumugam (2018)). Underlying many age-associated metabolic changes is a progressive deterioration in mitochondrial function (Gruber et al., 2013, 2008; Srivastava, 2017). Mitochondria are the primary producers of energy and play vital roles in maintaining health and homeostasis of cellular functions. It is, therefore, not surprising that mitochondrial dysfunction significantly contributes to aging and disease progression (Gruber et al., 2008; Srivastava, 2017). Specifically, a biproduct of mitochondrial respiration is the generation of reactive oxygen species (ROS), which under normal physiological conditions function in vital cell signaling. In excess however, increases in ROS drive pathophysiological processes such as protein and lipid oxidation that can induce senescence and accelerate disease pathology (Birla et al., 2020; Cherubini et al., 2020; Hall et al., 2015; Ricke et al., 2020; Saxena et al., 2019; Zhang et al., 2019).

An increase in ROS production from mitochondria is a known consequence of aging (Yonutas et al., 2015) and is further exacerbated by several neurodegenerative or neurotraumatic conditions (Birla et al., 2020; Cherubini et al., 2020; Pandya et al., 2007; Ricke et al., 2020). One way ROS is generated by mitochondria is when resistance to electron transfer increases, which induces slippage of electrons onto soluble oxygen forming the free radical superoxide (Suski et al., 2012; Zhao et al., 2019). Downstream reactive products of superoxide induce predictable and detectable cellular damage to lipids (4-hydroxynonenol, 4-HNE), proteins (3-Nitrotyrosine, 3-NT, when reacted with nitric oxide), and DNA, all of which accumulate with age and disease. Consequently, therapeutic strategies aimed at ameliorating mitochondrial dysfunction have emerged as promising approaches to mitigate or slow neurodegeneration occurring with age and disease or trauma (Caldeira da Silva et al., 2008; Yonutas et al., 2016). However, the extent to which mitochondrial changes with age contribute to, or confound, neurodegeneration and recovery is not well understood.

Increases in mitochondrial membrane potential are tightly associated with increased resistance to electron flow and accelerated ROS formation (Zhao et al., 2019). Under normal homeostatic conditions, increased ROS generated by mitochondria induce feedback regulation of mitochondrial regulatory proteins such as uncoupling proteins (UCPs) (Ježek et al., 2015; Pecqueur et al., 2001). UCPs reduce mitochondrial membrane potential, allowing flow of protons back into the mitochondrial matrix, effectively decreasing respiratory resistance and mitigating superoxide leak during electron transport (Zhao et al., 2019). Although decreasing mitochondrial ROS may appear beneficial to cellular health, increasing permeability of protons through the inner mitochondrial membrane uncouples respiration from ATP production (Geisler et al., 2016). In compromised environments such as in neurodegeneration or neurotrauma, mitochondria are therefore engaged in a competing balance to meet increased cellular energy demands while regulating ROS over-production. How this homeostatic imbalance is regulated with age in health and disease is not well understood. Based upon the potential double-edged sword of pharmacological uncouplers, in the current study we used the uncoupling agent 2,4-dinitrophenol (DNP) to study age-dependent mitochondrial function after spinal cord injury (SCI).

Mitochondria that have been injured following SCI increase ROS production which exacerbate neurodegeneration and tissue damage (Jin et al., 2004; McEwen et al., 2011; Pandya et al., 2007; Patel et al., 2009). This secondary injury after SCI can propagate for weeks-to-months following the primary insult. Mitigating respiratory ROS production immediately post-injury, or ROS generated from inflammatory non-mitochondrial derived sources, have proven effective and limits secondary injury (Impellizzeri et al., 2011; Jin et al., 2004; Khayrullina et al., 2015; Pandya et al., 2007; Patel et al., 2009; Zhang et al., 2019). How mitochondria contribute to a progressive neurodegeneration observed after SCI is less well understood, and almost nothing is known about how age effects this process. A lack of knowledge about how aging effects SCI is of growing concern as a shift in the average age at time of SCI has progressed from 28 years of age to 42 since the 1970’s (NSCISC, 2013). The prominent role of mitochondria in both aging and SCI has clinical implications since the efficacy of mitochondrial-based pharmacotherapies for neurotrauma may vary with age. This study utilized the progressive neurodegeneration occurring following SCI to interrogate how age affects mitochondrial regulation of secondary injury and recovery, using graded doses of DNP.

Collectively, we observed age-dependent effects on the function of isolated mitochondria after SCI and DNP treatment. *In vivo*, dose-response DNP treatment produced age-divergent effect on ROS accumulation, neurodegeneration, and functional recovery at multiple time points after SCI in 4- and 14-month old (MO) mice. Specifically, 14-MO mice benefitted from DNP treatment while DNP worsened outcomes in 4-MO mice. Collective results demonstrate that age-associated changes in mitochondrial function have profound effects on injury-induced neurodegeneration and highlight the importance of age as a biological variable in the treatment and progression of central nervous system disorders.

## 2. Material and methods

### 2.1. Animals, surgical procedures, and DNP delivery

All procedures followed institutional and national guidelines for the care and use of laboratory animals as approved by the University of Kentucky’s Animal Care and Use Committee. Female C57/Bl6 mice of age 4- (n = 63; Jackson Laboratories) and 14-months old (MO) (n = 67; National Institute for Aging) received surgical laminectomy followed by a 60 kdyn T9 contusion SCI (Infinite Horizons Impactor) as previously described (Zhang et al., 2016), or laminectomy only (n = 12). Mice of age 4- and 14-MO were chosen as the most phylogenetically comparable ages to represent the shift in clinical demographics from young adults to middle age at time of SCI (Dutta and Sengupta, 2016; Semple et al., 2013). Further, a 60 kdyn injury was chosen empirically based on previous work demonstrating that a 50 kdyn injury is too mild to sustain detectable functional deficits in 4-MO mice, while a 75 kdyn injury is too severe in 14-MO mice, resulting in increased mortality and an inability to weight support during objective functional analysis. Mice were anesthetized using intraperitoneal (IP) injections of ketamine (100.0 mg/kg) and xylazine (10.0 mg/kg), and received analgesic (Buprenex SR, 1.0 mg/kg day of surgery), antibiotic (Enrofloxacin, 5.0 mg/kg for 5 days), and saline (1.0 mL/day for 5 days) support following surgery. All mice were group housed for the duration of the study on a 14 h light/10 h dark cycle. Manual bladder evacuations were performed 2×/day for the duration of the study.

Mice were randomly assigned to receive DNP (Sigma Aldrich CO. St. Louis, MO; 1.0-, 2.5-, or 5.0-mg/kg), or vehicle (1% dimethyl sulfoxide; DMSO), which were delivered *via* intraperitoneal (IP) bolus injections (10 μL per g body weight) 30 min post-injury then daily for up-to 7 days. Stock 50.0 mg/mL concentrations of DNP were dissolved in DMSO and diluted in sterile phosphate buffered saline (0.01 M; PBS) immediately before use. Throughout the following three major experiments (1. *Ex vivo* mitochondrial analyses, 2. 7-day survival, 3. 28-day survival) a total of n = 4 4-MO mice, and n = 9 14-MO mice were lost due to premature morbidity or mortality or surgical complications. Respectively for 4- and 14-MO mice, n = 1- and 2-mice died overnight after SCI; n = 2- and 5- mice died under anesthesia for unknown reasons; n = 1- and 1-mice were excluded based upon *a priori* exclusion criteria for abnormal impact parameters indicative of a bone hit or spinal movement at the time of injury; and n = 0- and n = 1-mice were euthanized two days prior to the end of study due to a major open and untreatable skin wound; the spinal cord from this mouse was included in histology and did not vary from others in group. The cause of mortality remains unknown for the three mice that died overnight after receiving SCI. Two of the three mice received a 5.0-mg/kg dose of DNP, while the third received a 1.0-mg/kg dose, therefore toxicity from DNP may be possible. All other mice experiencing mortality under anesthesia died prior to delivery of DNP. Supplementary Table 1 contains lists of final sample sizes used for all groups in all outcomes.

### 2.2. Ex vivo mitochondrial response to DNP

Mitochondria were isolated as previously described (Brown et al., 2004) from 4- and 14-MO sham, or SCI-treated mice at 3- and 7-days post-injury (dpi). Briefly, mice were euthanized using CO_2_ followed by rapid decapitation. A 7-mm section of cord surrounding the lesion was quickly isolated and mechanically homogenized in respiration buffer. Tissue suspension was centrifuged at 1300 *g* to pellet debris, followed by collecting the supernatant and pelleting crude mitochondria at 13,000 g. Crude mitochondrial extract was suspended in respiration buffer and synaptic mitochondrial fractions were liberated using nitrogen bombing. Crude mitochondria were standardized based on protein content and evaluated for oxygen consumption rate (OCR) on the Seahorse (Seahorse XFe96 Analyzer; Agilent Technologies, Inc.; Santa Carla, CA) as previously described (Patel et al., 2009). The sensitivity of mitochondria to uncoupling was performed using pre-loaded auto-injection ports to administer 1-, 10-, or 100-μM DNP in series and the change in OCR was obtained. Next, to perform a mitochondrial stress test, mitochondria were isolated from sham or vehicle-treated SCI-mice, as well as SCI-mice treated with 1.0 mg/kg DNP for 3- or 7-dpi. All mitochondrial experiments were initiated by treating wells with 5 mM Pyruvate, 2.5 mM Malate, and 1.0 mM ADP to drive State III respiration. Mitochondrial stress tests proceeded as previously described (Sauerbeck et al., 2011), using sequential delivery of Pyruvate/Malate/ADP (State III), Oligomycin (State IV), FCCP (State V_CI_), and Rotenone/Succinate (State V_CII_) to test different respiratory states. Experiments were performed using 2–3 technical replicates per biological sample (n = 3/ group) depending upon auto-injecting success.

### 2.3. Functional analysis

Prior to SCI all mice were acclimated to testing paradigms including an open field for the Basso mouse scale (BMS; (Basso et al., 2006)) and the horizontal ladder (Cummings et al., 2007). Functional abilities were assessed after SCI using the BMS at 1-, 3-, 7-, 14-, 21-, and 28-dpi. Horizontal ladder was tested prior to injury and at 28-dpi for animals capable of performing at least coordinated stepping. This resulted in exclusion of 2 vehicle-treated 14-MO mice from analysis for an inability to step. To analyze horizontal ladder, video was captured from underneath as mice traversed a small 1.0 m ladder containing evenly spaced rungs. Misplaced hindlimb steps resulting in foot-slips beyond the plane of rungs were analyzed from video acquisition. Two reviewers who were blinded to experimental groups were used to assign BMS scores and one reviewer counted foot-slips on the horizontal ladder.

### 2.4. Histological outcomes

#### 2.4.1. Tissue preparation

At 7- and 28-dpi, vehicle and DNP-treated mice were anesthetized with a lethal dose of ketamine, exsanguinated using 0.1 M PBS, and perfused using 4% formaldehyde (Thermo Fisher Scientific, Waltham, MA). Spinal cord segments were collected, post-fixed in 4% formaldehyde for 2 h at room temperature, incubated overnight in 0.2 M phosphate buffer, then dehydrated with 30% sucrose (Sigma Aldrich CO., St. Louis, MO) at 4 °C. Spinal cords were placed side by side in cryomolds in sets of 4–5 cords/block in Optimal Cutting Temperature Compound (OCT), and frozen on dry ice. Spinal cords were arranged in a random order while ensuring at least one cord per group was frozen in each block. Sections were cut in the transverse plane at a 10-μm thickness and mounted in serial order, with 100 μm between serial sections on a slide. Prior to immunolabeling all slides were processed using heat-mediated antigen retrieval in 0.1 M sodium citrate buffer (pH 6.0) at 80 °C for 5 min.

Each set of immunolabeling, imaging, and analysis was performed for all animals in a single cohort, using identical staining, imaging acquisition, and analysis settings. Images were acquired using Zeiss Axioscan (model Z1, Carl Zeiss AG., Oberkochen, GE) and analyzed using Halo software (Indica Labs, Albuquerque, NM). Histology and immunohistochemistry was performed by individuals blind to group inclusion as follows.

#### 2.4.2. Tissue sparing and axon sprouting

Slides were stained with eriochrome cyanine (EC; Sigma Aldrich CO., St. Louis, MO) for myelin and counterstained with fast red (nuclei) at 7-dpi, or immunolabeled for axons using neurofilament 200 kD (1:2500; NFH; Aves Labs Inc., Davis, CA) at 28-dpi. Immunolabeling was visualized using 3,3′-diaminobenzidine (DAB; Vector Laboratories, Inc., Burlington, CA) as a chromogen. To analyze lesion areas, regions of spared tissue were traced, and areas were obtained. Tissue was considered spared based on macro structure morphology, evident by the clearly stained presence of white matter, or gray matter containing neurons and organized axonal architecture; tissue sections at the lesion epicenter typically have no spared gray matter and only a small rim of spared white matter. Spared tissue was assessed in each section to identify the section containing the least amount of spared tissue, which was used to define the lesion epicenter. All histological analyses were performed with respect to this defined epicenter. Due to a dynamic nature of lesion volumes at 7-dpi, the epicenter was used as a single predictor of tissue sparing for purposes of identifying an optimal dose for behavioral studies. At 28-dpi tissue sparing was analyzed every 100 μm for 700 μm rostral and caudal to the lesion. Tissue sparing at the lesion epicenter was compared between groups, as well as total sparing throughout all measured sections.

Axon sprouting was analyzed within the lesion at 28-dpi using a staining threshold to detect DAB labeled axon filaments. The average proportion of positive labeled pixels to lesion area was analyzed in 3 sections surrounding the epicenter per animal and used for comparison between groups.

#### 2.4.3. Reactive nitrogen species accumulation

Reactive nitrogen species (RNS) damage was assessed at 7-dpi using immunolabeling against 3-nitrotyrosine (3-NT; 06-284; 1:2000; Sigma Aldrich CO., St. Louis, MO), as a measure of protein nitration (Carrico et al., 2010). To analyze 3-NT labeling, tissue sections up to 1-mm rostral and caudal to the epicenter were traced and a threshold was created to exclude non-positively labeled pixels from analysis. A threshold for determining positive pixel density was set based on tissue containing no 3-NT labeling from a 4-MO mouse section. No 3-NT labeling was performed at 28-dpi as increases in 3-NT have been reported to return to near-baseline conditions before 14-dpi (Carrico et al., 2010).

#### 2.4.4. Neuronal survival

Neuronal sparing was assessed using immunolabeling against neuronal nuclei (NeuN; NBP1-77686; 1:4000; Novus Biologicals, Centennial, CO). Neuron viability was determined by tracing around the ventral horns of tissue sections and applying a particle counting algorithm available on the Halo software. Parameters were set to identify neurons based on density, circularity, size, and contrast. Neuron counts from every other section between 500 μm rostral and caudal to the epicenter was summed and used to analyze 7-dpi tissue, and every section between 500 μm rostral and caudal to the epicenter was summed for 28-dpi tissue.

### 2.5. Statistics

Statistical modeling was performed in collaboration with University of Kentucky’s interdepartmental Applied Statistics Laboratory (ASL). General linear models were created to determine the effects of age, dose, and injury when appropriate. When comparing between ages was appropriate two- or three-way analysis of variance (ANOVA) was used. This includes analysis of *ex vivo* mitochondrial experiments, proportional area of 3-NT labeling, and horizontal ladder using two-way ANOVA, as well as analysis of motor functions on BMS and BMS sub-scores using three-way ANOVA with repeated measures. Because spinal cord size increases with age, outcomes dependent on absolute tissue area (sparing) and cell counts (neuron survival) were analyzed using within age-group comparisons with one-way ANOVA or two-way repeated measures ANOVA when appropriate. To directly compare outcomes between ages, values of DNP treated groups were normalized to mean values of within-age vehicle treated groups and treatment effects were compared between ages using two-way ANOVA. Dunnett’s pair-wise comparisons were made to limit analysis within ages and between vehicle and DNP-treated groups. Sidak’s pair-wise comparisons were used to compare between-group treatment effects when normalized to vehicle controls. During analysis of neuron counts at 7-dpi, a single outlier was found using Grubbs’ analysis (value = 81; G = 1.737; *p* < 0.05) in the 4-MO 5.0-mg/kg/day DNP treatment group and was removed from analysis. No other outliers were identified. Statistical support was obtained from ASL to analyze BMS and BMS sub-scores due to the non-normal repeated measures nature of BMS sub-score data. BMS score models adjusted for the Day 1 score and BMS sub-scores were normalized by using a square root transformation of the change from Day 3. BMS analyses were completed using SAS 9.4 (SAS Institute, Cary, NC). All other analyses were completed using Prism v. 8.0 (Graphpad, San Diego, CA). Animal numbers for *in vivo* experiments were estimated by power analyses (1-β = 0.80; α = 0.05; one-way ANOVA) using standard deviations from pilot studies, as well as estimated effects from prior experiments in our lab investigating effects of age on treatment efficacy using antioxidant therapies (Zhang et al., 2019). For behavioral outcomes n = 10 was predicted sufficient to power BMS analyses, while n = 5 was estimated for histological tissue sparing. For *ex vivo* mitochondrial experiments, routine use of n = 3 with 3 technical replications has proven sufficient power in prior experiments from our group (Yonutas et al., 2015).

## 3. Results

### 3.1. Effects of mitochondrial uncoupling are age-dependent

**Fig. 1.**
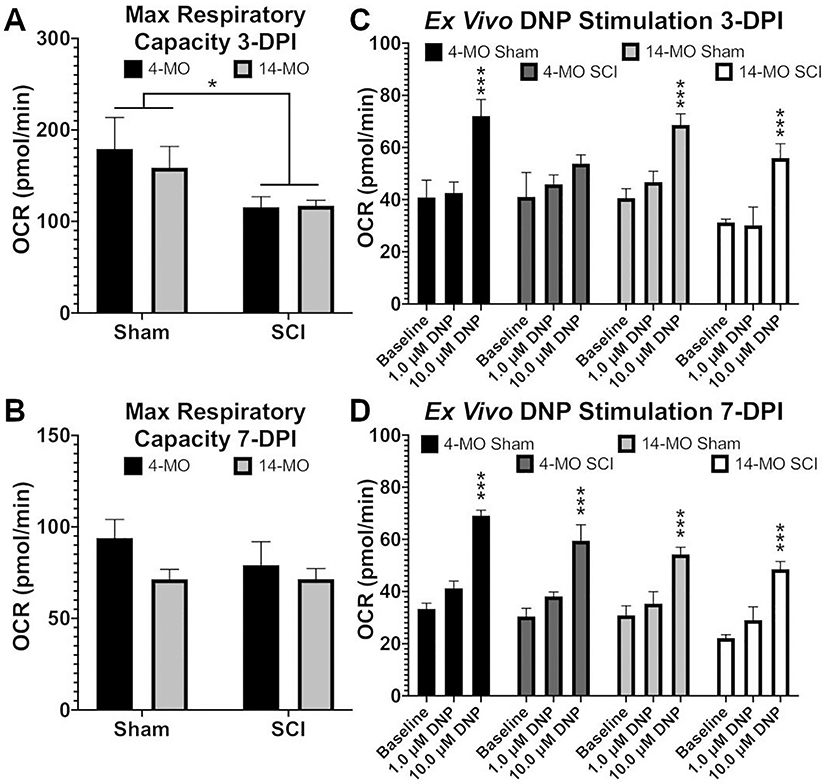
*Ex vivo* uncoupling of isolated mitochondria with DNP exert age-dependent effects. Mitochondria were isolated from 4- and 14-MO mice at 3- and 7-days post sham (laminectomy) or spinal cord injury (SCI). Graded doses of DNP were administered to determine the efficiency of generating an uncoupling response. At 3- days post-injury (DPI) maximum respiratory capacity was lower in mitochondria obtained from SCI− compared to sham-mice (A), no injury effect was detectable by 7-DPI (B). All groups exhibited a significant uncoupling response upon administration of 10 μM DNP at 3-DPI (*p* < 0.005) except mitochondria obtained from 4-MO SCI-mice (C). This age-dependent response was ameliorated by 7-DPI where all groups exhibited a significant uncoupling response upon administration of 10 μM DNP (*p* < 0.005; D). Analyzed using two-way ANOVA. Graphs represent mean ± SEM, n = 3/group. *p < 0.05, ***p < 0.005 *vs* vehicle (A, B) or *vs* baseline (C, D).

We first set out to determine if age and SCI affect mitochondrial function. Specifically, mitochondria were isolated from spinal cords with or without (sham) moderate thoracic T9 contusion (60 kdyn) SCI. OCR of isolated mitochondria was examined with the Seahorse bioanalyzer to determine effects of uncoupling using graded doses of DNP. High DNP doses (100.0 μM) were used to completely uncouple mitochondria, revealing significant SCI induced impairments in maximal respiratory capacity at 3- (F_(1,8)_ = 5.94, *p* < 0.05; Fig. 1A) but not 7-days post-injury (dpi; F_(1,8)_ = 0.66, *p* = 0.43; Fig. 1B). Surprisingly, age did not exacerbate mitochondrial dysfunction after SCI when completely uncoupled (Fig. 1). This SCI impairment of mitochondrial function is indicative of an impaired capacity for energy production that can last at least 3-, but not longer than 7-dpi.

Next we simulated mild uncoupling effects, which have been proven safe for use *in vivo* (Jin et al., 2004; Maragos et al., 2003; Patel et al., 2009), to determine if exposing mitochondria to low DNP doses (1.0–10.0 μM) were age- or injury-dependent. A significant uncoupling effect emerged at 10.0 μM DNP in mitochondria obtained from uninjured mice at both 3- (F_(2,4)_ = 24.86, *p* < 0.01; Fig. 1C) and 7-dpi (F = _(2,4)_ = 85.46, *p* < 0.005; Fig. 1D). After SCI however, only mitochondria isolated from 14-MO injured-mice were responsive to low DNP doses at 3 dpi (*p* < 0.005)(Fig. 1). By 7 dpi, all mitochondrial populations responded to low doses of DNP, confirming that injury induced deficits were no longer detectable (*p* < 0.005)(Fig. 1). Here we observed that mitochondrial responses to treatment after injury are age dependent and that older mitochondria surprisingly maintain a higher sensitivity to mitochondrial uncoupling. This age-dependent effect of isolated mitochondria to mild uncoupling led us to hypothesize that an optimal DNP dose for treating SCI *in vivo* may differ with age.

### 3.2. A low dose of DNP is toxic to 4-MO- but protective to 14-MO-SCI mice at 7-DPI

To determine if age-dependent uncoupling persists *in vivo* after SCI, we treated 4- and 14-MO SCI mice with graded doses of DNP for 7-dpi and examined outcomes associated with mitochondrial dysfunction: oxidative damage, neuron survival, and tissue sparing. Previous work from our lab has identified the greatest age-dependent accumulation of oxidative damage at 7-dpi (Zhang et al., 2016, 2015). Further, we found that 14-MO mice experience greater loss of tissue surrounding SCI lesions associated with this age-dependent increase in ROS (Zhang et al., 2019, 2016, 2015). For mid-thoracic SCI, tissue sparing surrounding the lesion strongly correlates to functional outcomes and therefore was investigated as a merit to an overall treatment effect of DNP (Basso et al., 1996).

**Fig. 2.**
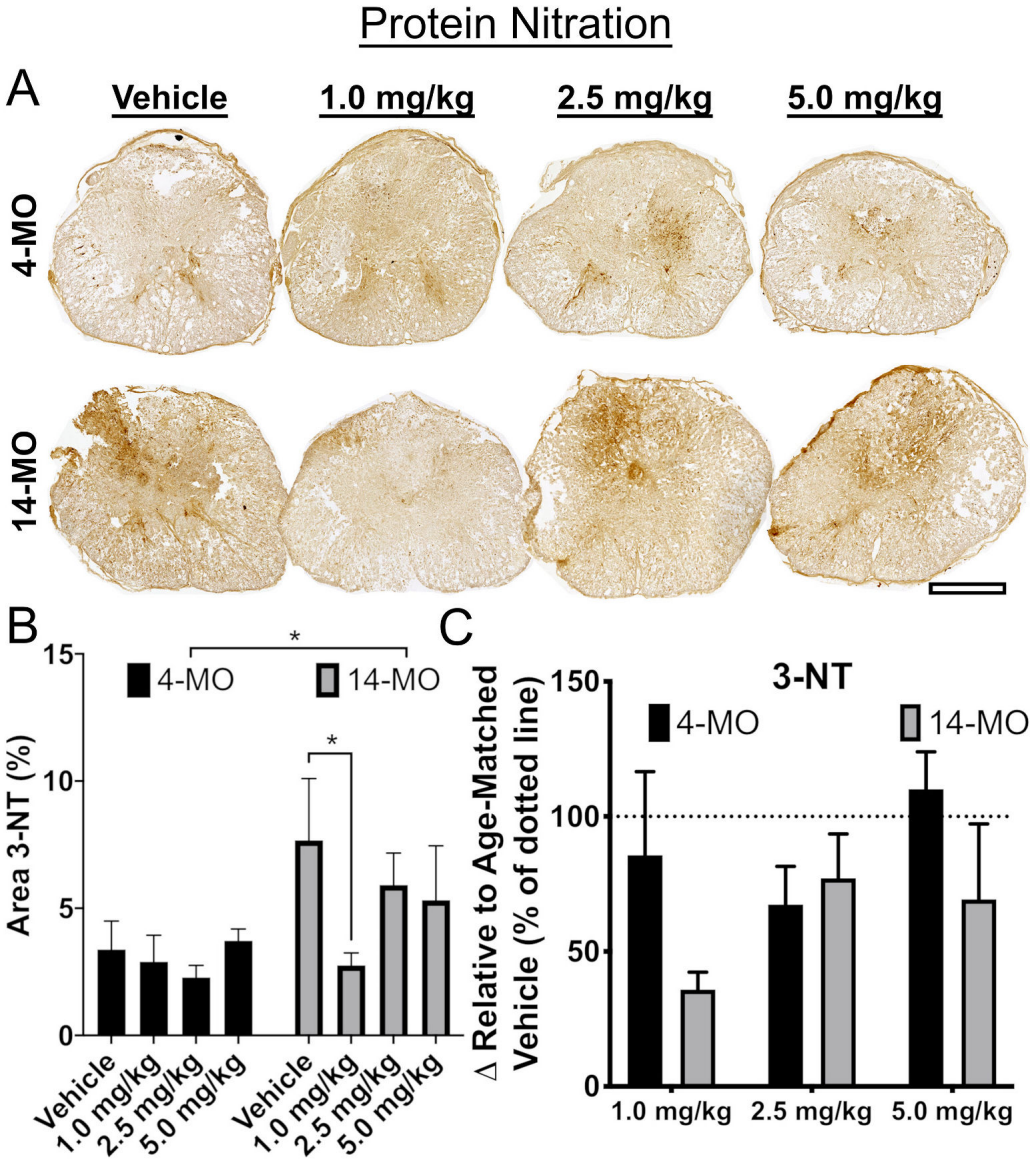
The biomarker of reactive oxygen species production, 3-NT, is reduced in 14-MO mice treated with DNP at 7 days after spinal cord injury. DNP was provided at 1.0-, 2.5-, or 5.0-mg/kg/day for 7 days to 4- and 14-MO mice after SCI to determine the effects of uncoupling on accumulation of oxidative stress. Immunohistochemistry was performed to label nitrotyrosine (3-NT) adducts that are indicative of upstream reactive oxygen species production. The 3-NT labeled tissue surrounding the lesion was analyzed as a percentage of the total cross sectional area of the cord. Images of representative samples 500 μm away from the lesion epicenter (A) reveal a significant increase in 3-NT expression in 14-MO mice after SCI (*p* < 0.05; B). A 1.0 mg/kg/day dose of DNP significantly reduced 3-NT accumulation only in 14-MO mice after SCI (*p* < 0.05; B). When compared to vehicle-treated mice, no significant differences were found in a treatment effect with DNP between 4- and 14-MO mice (*p* = 0.09; C). Scale bar represents 500 μm. Analyzed using two-way ANOVA. Graphs represent mean ± SEM, n = 3-5/group. *p < 0.05.

To delineate the contribution of mitochondrial-derived ROS to exacerbated tissue loss with age, we evaluated a dose-dependent response of mild-uncoupling using DNP for 7-dpi. DNP doses were chosen in accordance with prior literature citing a therapeutically effective range from 1.0- to 5.0-mg/kg in young rodents (Maragos et al., 2003; Patel et al., 2009). Consistent with our previous work finding increased ROS damage (4-HNE) in 14- compared to 4-MO mice at 7 dpi (Zhang et al., 2016), here we found an age-dependent increase in 3-NT accumulation (F_(1,30)_ = 5.31, *p* < 0.05; Fig. 2B). Further, we found that the lowest DNP dose (1.0-mg/kg/day) significantly reduced 3-NT accumulation only in 14-MO mice relative to vehicle controls (*p* < 0.05; Fig. 2B), demonstrating consistency with *ex vivo* analyses indicating that 14-MO injured-mitochondria are responsive to low DNP doses (Fig. 1). DNP treatment was ineffective at reducing oxidative damage in 4-MO mice regardless of dose (Fig. 2B). When results from 14- and 4-MO mice were compared directly by normalizing to vehicle controls within each age, no significant effects were found (Fig. 2C).

**Fig. 3.**
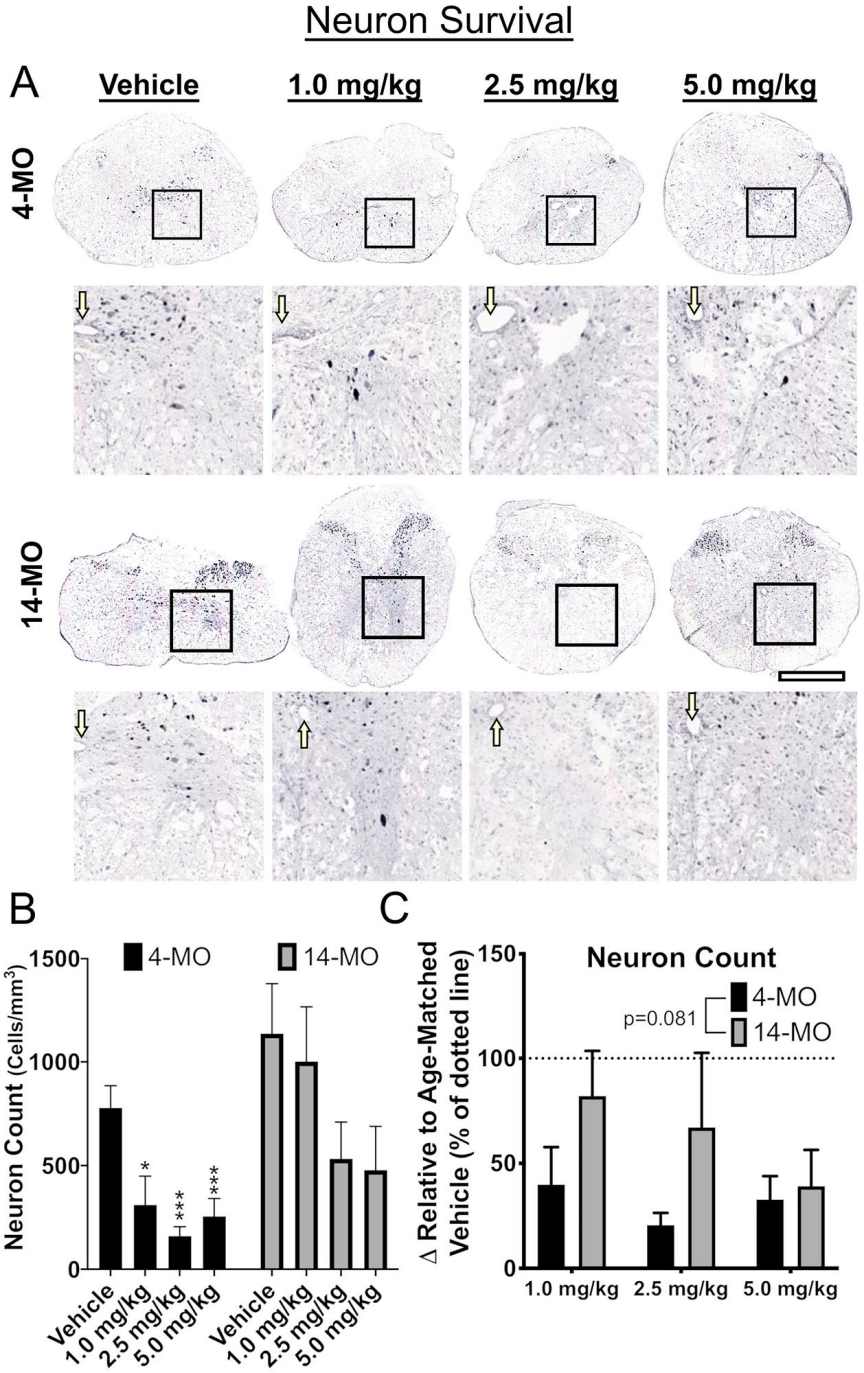
DNP exerts toxic effects on neuron survival in 4-MO mice at 7 days after spinal cord injury. Tissue obtained from mice at 7-DPI was analyzed for effects of DNP on neuron survival. Images of representative samples taken at 500 μm away from the lesion epicenter (A) demonstrate a significant loss of neurons with all DNP doses in 4- but not 14-MO mice (*p* < 0.05 for 1.0- and *p* < 0.005 for 2.5- and 5.0-mg/kg; B). When compared to vehicle-treated mice, no differences were found in a treatment effect with DNP between 4- and 14-MO mice (*p* = 0.08; C). Scale bars represent 500 μm. Boxes represent analysis regions over the ventral horns, and enlarged images. Arrows point to the central canal. Analyzed using one-way ANOVA for each age (B) or two-way ANOVA (C). Graphs represent mean ± SEM, n = 3-5/group. *p < 0.05, ***p < 0.005 *vs.* vehicle control.

**Fig. 4.**
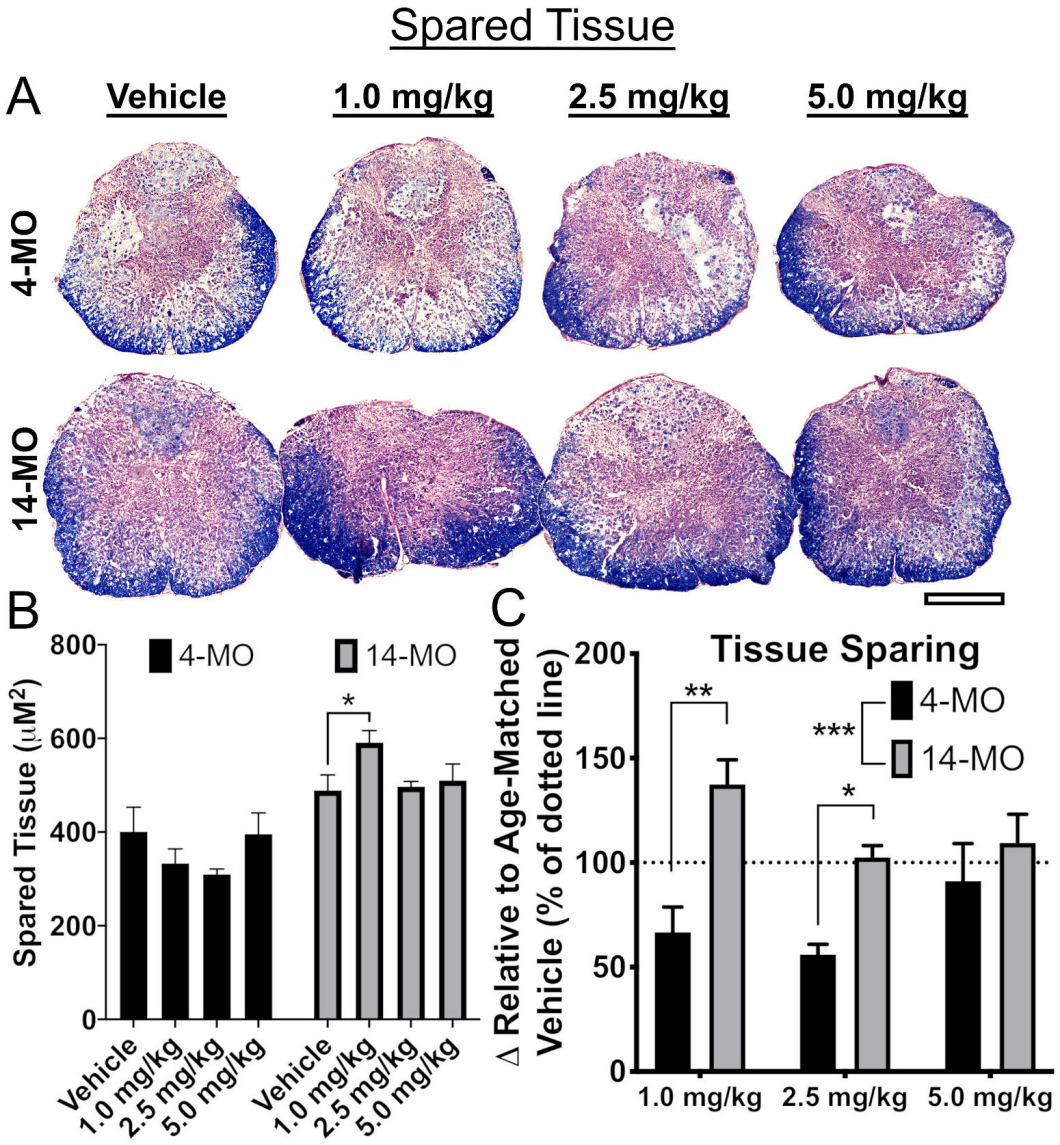
DNP exerts age-divergent effects on tissue sparing at 7 days after spinal cord injury. The extent of tissue sparing was assessed at 7-DPI after treatment with DNP. Images of representative samples of lesion epicenters (A) demonstrate that treatment with 1.0-mg/kg/day DNP spared tissue in 14- but not 4-MO mice treated (*p* < 0.05; B). When compared to same age vehicle-treated groups, the 1.0- and 2.5-mg/kg/day DNP doses resulted in significant age divergent responses by reducing tissue sparing in 4-MO SCI mice and preserving tissue in 14-MO SCI mice (C). Scale bars represent 500 μm. Analyzed using one-way ANOVA for each age (B) or two-way ANOVA (C). Graphs represent mean ± SEM, n = 3–5/group. *p < 0.05, **p < 0.01, ***p < 0.001.

Next, effects of DNP treatment on neuron survival and tissue sparing were assessed within each age. For neuron sparing, 14-MO SCI mice treated with DNP displayed no overall effects (F_(3,15)_ = 2.05, *p* = 0.15; Fig. 3B). Higher doses trended towards neuron toxicity but did not reach significance (*p* = 0.14; Fig. 3B). In contrast, spinal cords from 4-MO mice treated with DNP displayed an acute toxic response apparent through a significant loss of neurons surrounding the lesion (F_(3,12)_ = 9.16, *p* < 0.005) at all DNP doses (*p* < 0.05; Fig. 3B). There were dose-dependent differences in tissue sparing in 14-MO SCI mice (F_(3,16)_ = 2.88, *p* = 0.06; Fig. 4B) with the 1.0-mg/kg/day dose varying significantly from vehicle (Dunnett’s pairwise comparison, *p* = 0.05). There was no effect of higher DNP doses on tissue sparing in 14-MO mice (*p* = 0.92), and no effects on spared tissue were found in 4-MO mice (F_(3,14)_ = 1.21, *p* = 0.34; Fig. 4B).

Comparing the absolute values of neuron survival and tissue sparing between 4- and 14-MO mice is technically inappropriate because the spinal cord size increases with musculoskeletal maturity over the first 7–8 months of age (Quinn, 2005). This can make an increased absolute area of spared tissue in 14-MO, relative to 4-MO mice, proportionally less compared to pre-injury values. Comparing tissue sparing between ages is therefore compromised due to inherent size differences of the spinal cord. An apparent increase in neurons surrounding the lesion in 14-MO mice was, however, unexpected. We do not yet know if this is a biological response or a technical artifact similar what was described for tissue sparing, however, this effect was reproduced at 28-DPI and can be found below (Fig. 6).

In consideration of the technical limitations of directly comparing effects of DNP on neuron survival and tissue sparing between ages, we normalized values to age-matched vehicle-treated controls. We observed no significant effects (F_(2,19)_ = 3.38, *p* = 0.08; Fig. 3C) of age on neuron toxicity but did observe a significant age-divergent response to DNP treatment in spared tissue surrounding the lesion (F_(1,21)_ = 13.72, *p* < 0.001; Fig. 4C). *Post hoc* analyses revealed a significant increase in tissue sparing in 14- compared to 4-MO mice treated with 1.0- (*p* < 0.01) and 2.5-mg/kg/day DNP (*p* < 0.05) relative to their respective vehicle-treated controls (Fig. 4C). Together, these data support our *ex vivo* findings of mitochondria from 14-MO SCI-mice being more responsive to mild uncoupling. Further, our data demonstrate that age has a profound effect on mitochondria-mediated neurodegeneration as indicated by opposite treatment effects in 14- and 4-MO SCI-mice.

## 4. DNP maintains age-divergent effects at 28-DPI

Due to fluctuating events acutely within the injured microenvironment, recovery from injury can take weeks to stabilize (Basso et al., 2006; Detloff et al., 2012; Silva et al., 2014). Previously we reported that acute mitigation of ROS production in 14-MO SCI-mice leads to improved and stabilized recovery by 3–4 weeks post-injury (Zhang et al., 2019). Therefore, we examined functional and anatomical recovery for 28-dpi to determine long-term effects of DNP treatment on SCI neuropathology and functional outcomes. Because data obtained at 7-dpi was inconsistent with prior literature that suggests a 5.0-mg/kg/day dose of DNP is most protective (Jin et al., 2004; Patel et al., 2009), we utilized both a 5.0- and 1.0-mg/kg/day dose that appeared most efficacious in 14-MO mice.

**Fig. 5.**
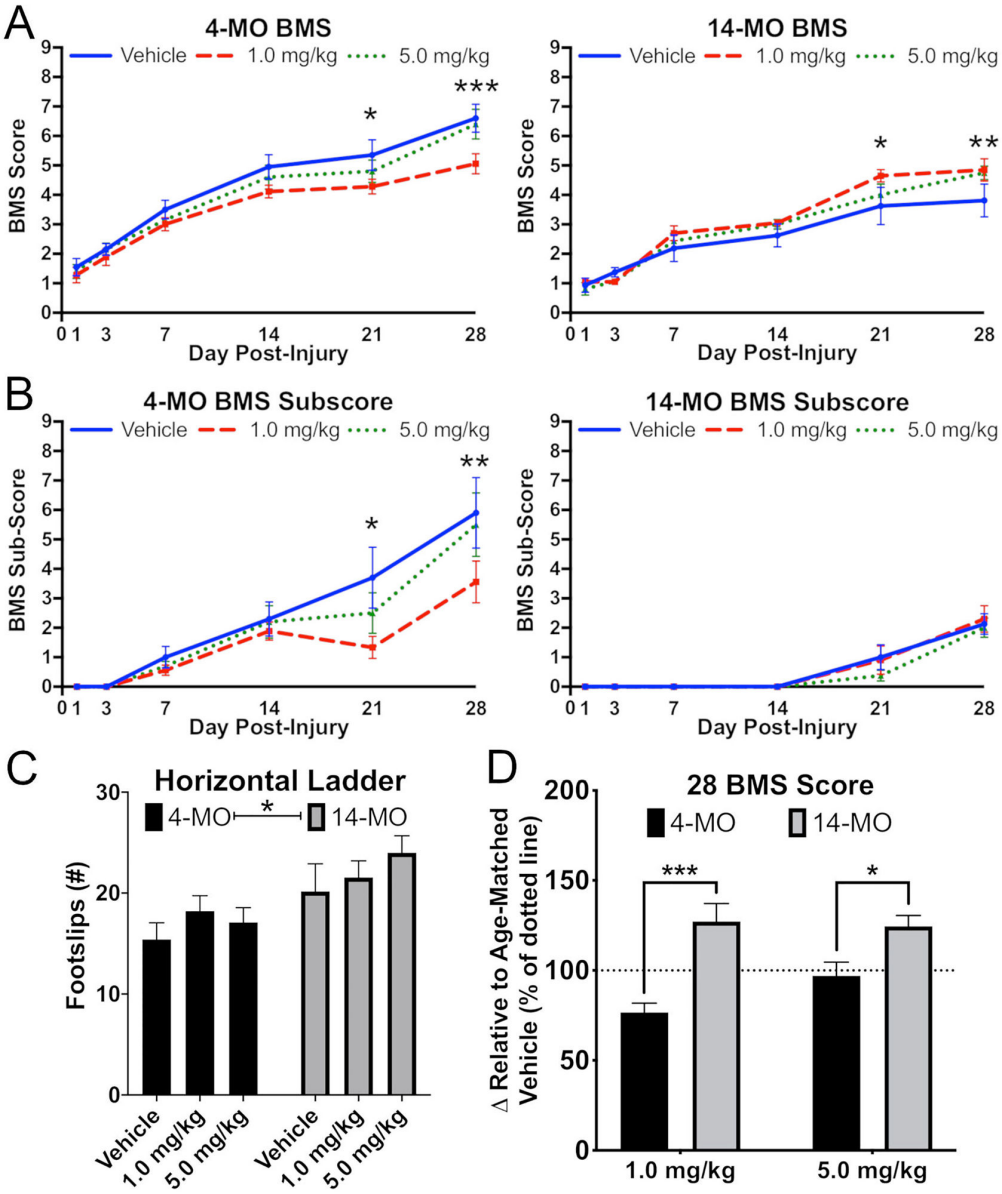
DNP exerts age-divergent effects on motor functions after spinal cord injury. DNP treatment (1.0-mg/kg/day for 7 days after injury) significantly reduced motor function by 28-DPI in 4-MO SCI mice (*p* < 0.005) but improved recovery in 14-MO SCI mice (*p* < 0.05) when analyzed with BMS motor score (A,D). A significant improvement in BMS scores was also observed in 14-MO, but not 4-MO, mice treated with 5.0-mg/kg/day (*p* < 0.05; A). Similar effects were observed for BMS sub-scores in 4- but not 14-MO mice by 28-DPI (*p* < 0.01; B). A main effect of age was observed for foot-slips on horizontal ladder (*p* < 0.005) but no effects were found for treatment with DNP (C). Age-divergent effects were apparent at 28-DPI on BMS scores when compared to age-matched vehicle-treated mice at both 1.0- (*p* < 0.005) and 5.0 mg/kg/day doses (*p* < 0.05; D). Analyzed using Three-way ANOVA with repeated measures (A, B) or two-way ANOVA (C, D). Graphs represent mean ± SEM, n = 8-10/group for BMS and BMS sub-score and n = 6–10/group for horizontal ladder. *p < 0.05, **p < 0.01, ***p < 0.005, *vs* same day vehicle controls.

As reported previously, we observed a significant effect of age on locomotor outcomes after SCI with 14-MO mice having significantly impaired over ground (BMS; F_(1,49)_ = 15.18, *p* < 0.0005; Fig. 5A) and proprioceptive (horizontal ladder; F(2,47) = 12.21, *p* < 0.001; Fig. 5C) locomotor function relative to 4-MO mice (Zhang et al., 2019, 2016, 2015). There were no interactions among treatment and age on the horizontal ladder. A significant three-way interaction (F_(8,199)_ = 2.01, *p* < 0.05) was found for motor recovery on open field BMS analysis, suggesting a differential response to DNP with age (Fig. 5A). Specifically, 14-MO mice treated with either 1.0- or 5.0-mg/kg/day had significant improvements in overt locomotion (BMS scores; *p* < 0.05) compared to vehicle treated mice. In contrast, 4-MO mice treated with 1.0-mg/kg/day DNP demonstrated toxicity apparent through significantly worse motor recovery by 21-dpi in measures of both overt locomotion (BMS; *p* < 0.005) and finer details of hindlimb and locomotor function (BMS sub-scores; *p* = 0.01; Fig. 5B). Similar to data obtained at 7-dpi, significant age-divergent effects were apparent in BMS scores (F_(1,34)_ = 10.44, *p* < 0.01) for mice treated with 1.0-mg/kg/day of DNP (*p* < 0.005) after normalizing values to age-matched vehicle-treated mice (Fig. 5D). Specifically, on average 14-MO SCI-mice improved by >25% relative to vehicle-treated mice, while locomotor function in 4-MO SCI mice decreased by ~25% (Fig. 5D).

**Fig. 6.**
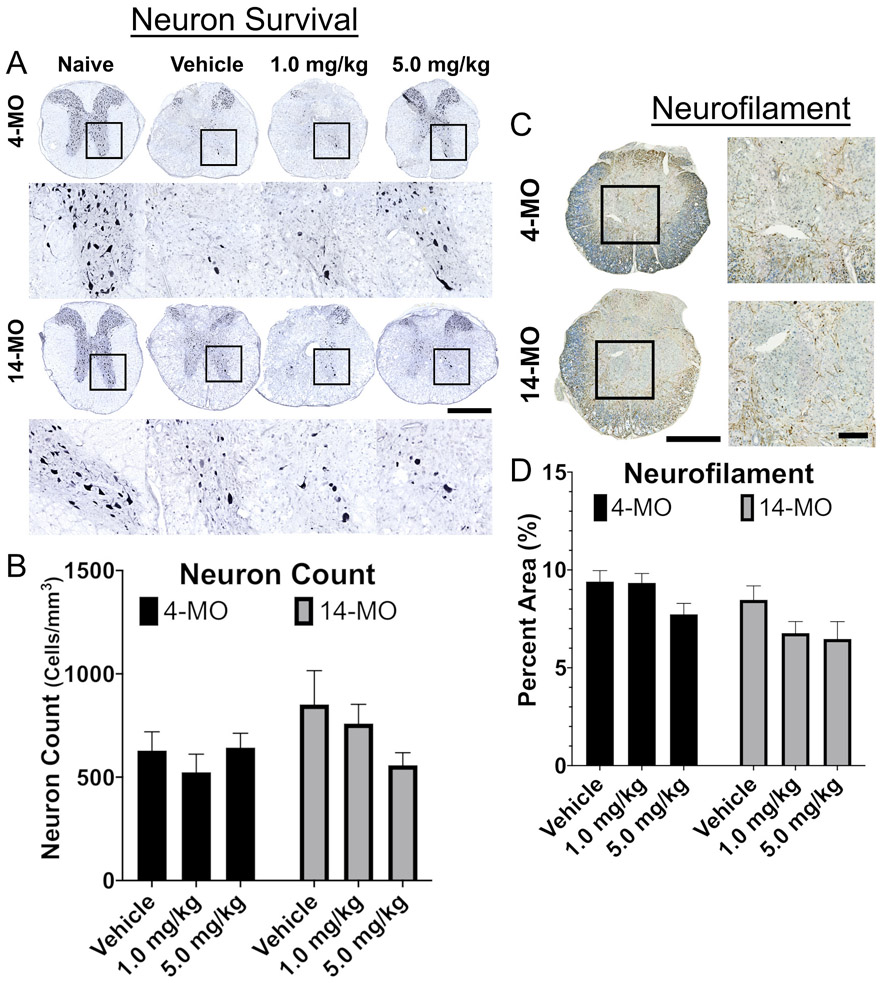
DNP does not exert significant effects on neuron survival or axon sprouting at 28 days after spinal cord injury. Toxic effects of DNP on neurons were normalized by 28-DPI, similarly no significant effects were found for axon sprouting. Images of representative samples obtained at 500 μm rostral to the lesion epicenter (A) or at the lesion epicenter (C) demonstrate no differences in neuron survival (B) or axon sprouting in the lesion (D). Scale bars represent 100 μm (C) and 500 μm (D). Analyzed using one-way ANOVA for each age. Graphs represent mean ± SEM, n = 8–10/group.

**Fig. 7.**
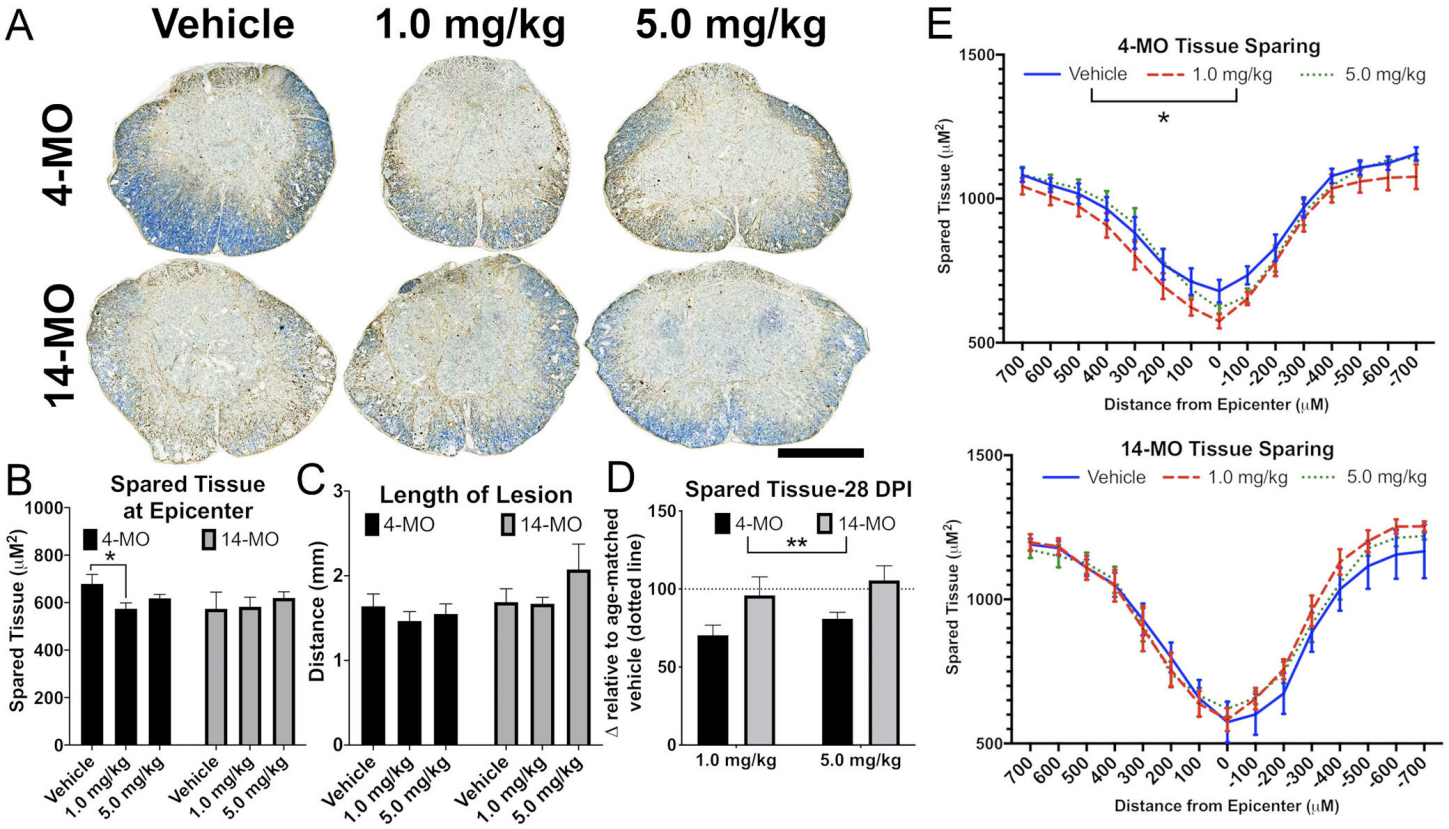
DNP reduces tissue sparing in 4-MO mice at 28 days after spinal cord injury. Lesion sizes stabilize by 28-DPI, which revealed a sustained toxic effect of DNP on tissue preservation to 4-MO mice, with no effects occurring in 14-MO mice. Lesion epicenter images representative of group means (A) demonstrate a significant reduction in tissue sparing in 4- but not 14-MO SCI mice treated with 1.0-mg/kg/day (for 7 days after SCI) of DNP (B). Treating SCI mice with 1.0-mg/kg/day of DNP reduced spared tissue spanning the lesion up to 700 μM rostral and caudal to the epicenter in 4-MO animals compared to vehicle-treated controls (*p* < 0.05). Overall effects of DNP treatment exerted an age-dependent toxic response that reduced tissue sparing significantly in 4-MO mice with no effects on 14-MO mice (*p* < 0.01; D). Scale bars represent 500 μm. Analyzed using one-way ANOVA for each age (B,C), two-way ANOVA (D), or two-way ANOVA with repeated measures for each age (E). Graphs represent mean ± SEM, n = 8–10/group. *p < 0.05, **p < 0.01.

With regards to long term neurodegenerative outcomes, no significant effects were detected for treatment on neuron survival (4-MO F_(2,26)_ = 0.59, *p* = 0.55; 14-MO F_(2,24)_ = 1.56, *p* = 0.23; Fig. 6A, B), or axon sprouting at 28-dpi (4-MO F_(2,26)_ = 3.17, *p* = 0.058; 14-MO F_(2,24)_ = 2.22, *p* = 0.12)(Fig. 6C, D). Not observing a similar toxic effect on neurons at 28-dpi, as we did at 7-dpi, suggests that DNP accelerated the death of neurons that were likely going to experience a delayed death after injury alone. However, locomotor recovery after SCI is more closely associated with the extent of tissue sparing surrounding the lesion, particularly at the lesion epicenter (Basso et al., 1995). Consistent with our observations of age-divergent effects of DNP treatment on locomotor function, we observed that DNP exerts an overall toxic effect on tissue sparing at the lesion epicenter in 4- (F_(2,26)_ = 3.804, *p* < 0.05) but not 14-MO SCI-mice (F_(2,24)_ = 0.11, *p* = 0.89; Fig. 7B). Specifically, DNP exacerbated tissue loss at the lesion epicenter in 4-MO mice treated with 1.0- (*p* < 0.05), but not 5.0-mg/kg/day (*p* < 0.15; Fig. 7B), as well as, an overall loss of tissue extending 700 μm rostral and caudal to the epicenter (*p* < 0.05)(Fig. 7). No differences were found for the rostral-caudal length of lesion (4-MO F_(2,26)_ = 0.46, *p* = 0.63; 14-MO F_(2,24)_ = 1.46, *p* = 0.25; Fig. 7C), or when evaluating tissue sparing with repeated measures over a 7 mm span of sectioned tissue (4-MO F_(2,26)_ = 1.464, *p* = 0.25; 14-MO F_(2,24)_ = 0.27, *p* = 0.76) in either age (Fig. 7E).

Comparing spared tissue in DNP-treated mice relative to vehicle-treated mice between ages revealed that DNP exerts an overall significant age-dependent toxicity to 4-MO mice (F_(1,33)_ = 8.79, *p* < 0.01), with ~25% decrease in tissue sparing with each dose (1.0-mg/kg/day, *p* = 0.07; 5.0-mg/kg/day, *p* = 0.09) (Fig. 7D). DNP treatment did not vary significantly from vehicle controls in 14-MO SCI mice. Together with our data from 7-dpi, our results show that acute age differences in mitochondrial responses to injury manifest in long term functional deficits and neurodegeneration in an age-divergent manner.

## 5. *In vivo* DNP significantly reduces complex I mediated respiration in aged mice

Having replicated age-divergent effects of treating SCI-mice with 1.0 mg/kg DNP at both 7- and 28-dpi, we sought to investigate how *in vivo* DNP treatment affects mitochondrial functions. We isolated mitochondria from 4- and 14-MO mice receiving either sham-, SCI + vehicle, or SCI + 1.0 mg/kg/day DNP for either 3- or 7-days after SCI. Seahorse was used to determine respiratory efficiency in State III and State IV respiration, as well as to test for complex I and II mediated electron transport. ATP-linked respiration was calculated by subtracting State III from State IV respiration to control for non-respiratory oxygen consumption. Coupling efficiency was also calculated using respiratory control ratios (RCR; State III/State IV).

**Fig. 8.**
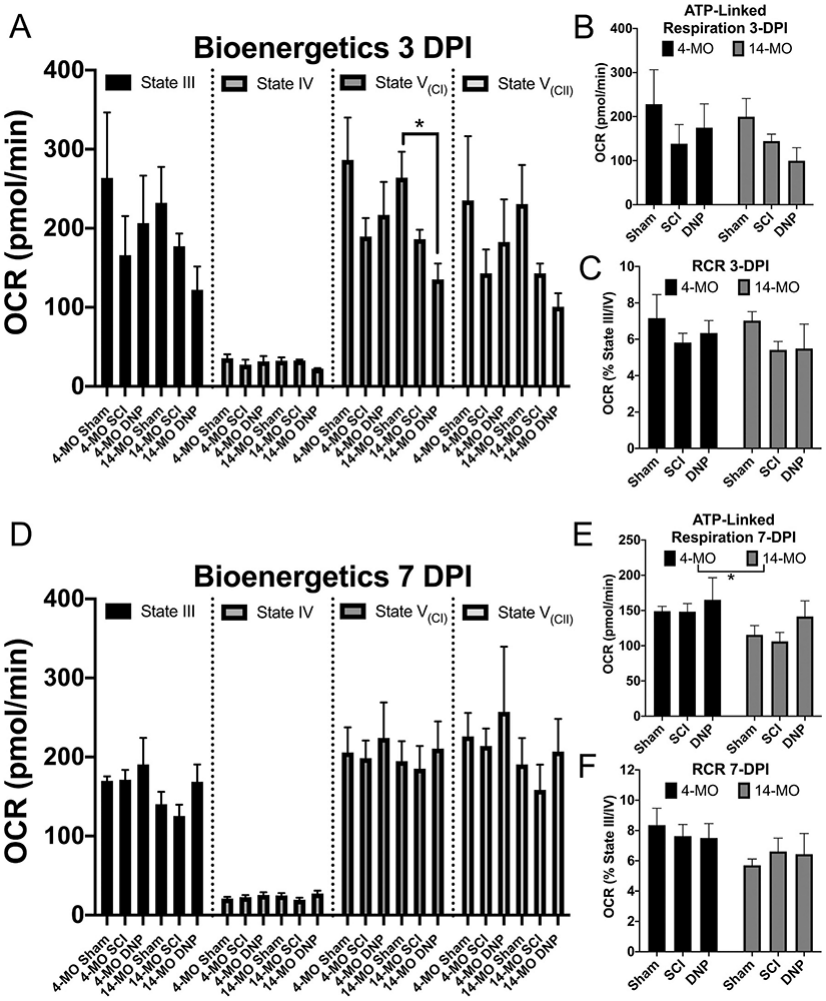
DNP lowers complex I-mediated electron transport with a preserved coupling efficiency in 14-MO mitochondria at 3-DPI. Mitochondria were isolated from mice receiving sham-, SCI−, and SCI + 1.0 mg/kg/day DNP at 3- (A–C) and 7-DPI (D–F), and evaluated for bioenergetic capacity using a mitochondrial stress test on Seahorse. The difference between mitochondrial state III and IV was used to evaluate ATP-linked respiration (B, E) and the ratio was used to calculate RCR as a measure of coupling efficiency (C, F). Complex I and II mediated electron transport were determined during State V respiration. DNP significantly impaired complex I mediated electron transport relative to sham-controls in 14-MO mice (*p* < 0.05), with SCI-only exerting an insignificant intermediate effect (*p* = 0.26 vs sham). DNP exerted insignificant trends towards an overall reduction in oxygen consumption and ATP-linked respiration further away from sham values relative to SCI-only at 3-DPI in 14-MO mice, however, DNP did not further reduce RCR coupling efficiency. DNP treatment or SCI-alone exerted no significant effects at 7-DPI, but main effects of age reduced ATP-linked respiration (*p* < 0.05). Each outcome was analyzed using two-way ANOVA with Tukey’s post hoc used for pairwise comparisons within each age. Graphs represent mean ± SEM, n = 3/group.

While no significant interactions were found, effects of DNP on mitochondrial functions persisted to display an age divergent trend at 3-DPI, only in an unexpected direction. DNP appeared to further exacerbate SCI-induced mitochondrial deficits in 14-MO mice, and significantly impaired complex I mediated electron transport compared to sham controls (*p* < 0.05; Fig. 8A). No other significant effects were found at 3-dpi (Fig. 8A-C) and no injury or treatment effects were observed at 7-dpi (Fig. 8D-F), however, ATP linked-respiration was decreased as a main effect of age as previously reported (F_(1,12)_ = 4.97, *p* < 0.05; Fig. 8D; (Yonutas et al., 2015)). While the implications of a reduced complex I capacity may appear detrimental at first, this may result in less produced ROS from a lowered metabolic activity. This would be consistent with our findings that 1.0 mg/kg/day DNP attenuated 3-NT accumulation in 14-MO mice.

## 6. Discussion

As life expectancies increase, age-associated declines in cellular processes play an increasing role in the pathophysiology of neurodegenerative conditions. Interactions between how age affects tissue responses in injury and disease will likely have unique consequences on therapeutic strategies. Without considering age as a variable in clinical or pre-clinical studies, it will be difficult to predict how biological processes respond to insults across the lifespan. Neurotrauma is specifically affected by age, as traumatic brain injury (TBI) exacerbates age-associated neurodegeneration (*i.e.* chronic traumatic encephalopathy) and the demographic of spinal cord injury (SCI) is shifting to older individuals sustaining injuries.

The major aim of this study was to determine how aging effects mitochondrial regulation of neurodegeneration in a mouse model of SCI. We found that mild mitochondrial uncoupling exerts age-divergent effects, ultimately resulting in improvements in function in 14-MO (month old) SCI-mice but further impairing function of 4-MO SCI-mice. Specifically, we observed that after SCI, age increases the sensitivity of isolated mitochondria to *ex vivo* uncoupling using DNP. *In vivo*, we observed that DNP reduced neuron and white matter survival in 4-MO SCI-mice, but the same dose had protective effects in 14-MO SCI-mice and reduced protein nitration. This age-divergent response to DNP manifested as long-term impairments in tissue preservation and locomotor function in 4-MO SCI-mice with contrasting improvements in 14-MO mice. Finally, *in vivo* treatment with DNP reduced complex I mediated electron transport only in isolated 14-MO injured mitochondria, but preserved coupling efficiency (RCR) relative to mice receiving SCI and vehicle. A decrease in respiratory capacity when RCR is preserved can be indicative of lower global electron transport activity. Under these conditions’ mitochondria may reduce ROS production, which might underly why DNP decreased 3-NT accumulation only in 14-MO mice. Our findings point to the endogenous regulation of mitochondrial function as an important mediator of recovery at younger ages that shifts towards a contributor of pathology with advanced age. Further, our findings emphasize a need to consider the age dependence of therapeutic interventions targeting SCI as well as various other neurodegenerative conditions.

As knowledge of neurological disorders advance, diagnostic capabilities are becoming available at earlier stages of disease. Moving forward it will be of vital importance to consider age at the time of treatment as a variable in our efforts to advance personalized medicine. If similar interactions exist in other neurological conditions or with different drugs, our data indicate that intervention at too early an age can possibly accelerate disease progression. Researchers often utilize accelerated animal models of disease such as the 3xTg mouse model of Alzheimer’s disease, the R6/2 model of Huntington’s disease, or the 6-hydroxydopamine model of Parkinson’s disease to understand neurological conditions of aging (Hernandez-Baltazar et al., 2017; Li et al., 2005; Sterniczuk et al., 2010). Our findings indicate that interactions between an animals age and induced onset of disease may confound interpretations of how mitochondria contribute to pathogenesis and challenge the translatability of treatment strategies into clinical populations. In particular, discontinuities in ages used for pre-clinical stroke studies compared to patients enrolled in clinical trials is increasingly implicated in the widespread failure to translate therapies (Ay et al., 2005; Badan et al., 2003; Cheng et al., 2009; Herson and Traystman, 2014; Pennypacker et al., 2017).

Within the field of neurotrauma our findings have far reaching implications. First, clinical demographics are progressing towards an increased average age at time of SCI (NSCISC, 2013; Toda et al., 2018). Yet, when evaluating the therapeutic potential of drugs in clinical populations, age is often not considered as a variable underlying treatment efficacy. To date the only drug used to treat SCI is methylprednisolone, which functions predominately to suppress injury-induced inflammation (Bracken et al., 1990, 1997). Efficacy of methylprednisolone as a treatment for SCI has been under scrutiny and is falling out of use due to complications associated with immune suppression. However, advancements in rodent models of SCI have recently demonstrated that treatments targeting ROS and inflammation may exert effects in older rodents even when little effects are found at younger ages (Zhang et al., 2019). This should merit re-evaluation of clinical data to determine if the effectiveness of previously investigated drugs targeting ROS exert improved efficacy with age (Stewart et al., 2019).

Given the resilience of younger individuals to neurological recovery after trauma or disease, one would predict that an increased age at time of injury would decrease treatment-mediated recovery. Unexpectedly, in the current study, we found that age increased the sensitivity of isolated injured mitochondria to *ex vivo* uncoupling with DNP, and found that DNP exerts reciprocal effects to 4- and 14-MO mice after SCI. As we and others have demonstrated, this may be due to ROS playing a larger role in the pathophysiology of SCI with advanced age (von Leden et al., 2017; Zhang et al., 2016). Due to the juxtaposing nature of mitochondrial uncoupling by decreasing both ROS and ATP, what can be derived from our work is that net contributions of mitochondrial function after SCI progress towards a more pathological role with advancing age in part by increasing free radical damage.

The mechanisms underlying the age-divergent effects found in this study remain unknown. While cellular toxicity caused by decreased ATP is possible, there is little evidence to suggest that mild uncoupling using doses in this study would exert a major energy crisis. Rodent studies in Huntington’s disease have used repeated dosing of DNP at 1.0 mg/kg/day and found beneficial effects on the progression of the disease (Wu et al., 2017); however, repeated dosing at 5.0 mg/kg did inevitably result in toxic effects. This contrasts our study that demonstrated the higher 5.0 mg/kg dose of DNP was less toxic than a lower 1.0 mg/kg dose to 4-MO mice. Since DNP uncouples mitochondria in a dose-dependent manner (Fig. 1), and a higher dose of DNP was less toxic in our study, there are likely alternative mechanisms to a major energy crisis that can explain the observed age-divergent trends.

One possible mechanism to explain our age-divergent effects may be attributed to a decreased calcium uptake by mitochondria treated with DNP (Sedlic et al., 2010). Glutamate-induced excitotoxicity occurring after SCI induces high cellular influx of Ca^2+^ which is buffered by mitochondria. At a certain Ca^2+^ influx threshold, the mitochondrial permeability transition pore (mPTP) opens and floods the cytoplasm with Ca^2+^, collapses the membrane potential, and expels NAD(H) (Nicholls and Ward, 2000). Whereas transient opening of the mPTP can be a physiological regulator of ROS production, sustained opening of the mPTP swells the outer mitochondrial membrane, causing release of cytochrome C into the cytoplasm and initiation of apoptotic cascades (Huang et al., 2001). Our data found that older and injured mitochondria uncouple at lower DNP doses, which may confer a greater resistance to mitochondrial Ca^2+^ uptake in 14-MO SCI-mice. Under these circumstances, any modest toxicity associated with decreased ATP production may be counter-balanced by a therapeutic capacity to reduce Ca^2+^ uptake and limit opening of the mPTP.

A decreased Ca^2+^ uptake by mitochondria can result in greater cytosolic Ca^2+^ and lead to downstream activation of transcriptional activity. Additionally, while Ca^2+^-induced gene regulation is probable following DNP administration, effects of DNP have been affiliated with upregulated cAMP-associated genes independent of Ca^2+^ signaling (Sebollela et al., 2010). Effects found by Sebollela et al. (2010) are particularly intriguing because the *in vitro* DNP doses used were low enough to not impact mitochondrial oxygen consumption. This demonstrates that DNP doses in the mild-uncoupling range can exert off-target effects. Due to the function of DNP as being both a weak acid and very membrane permeable, it is likely that other organelles that function on pH gradients, such as lysosomes, may be affected by mitochondrial uncouplers (Andres et al., 1996). In the context of aging, lysosome dysfunction has been affiliated with detrimental autophagic abilities (Mattson and Arumugam, 2018). If DNP does exert effects on lysosomal function, it may be possible for sustained delivery of DNP to exert age-divergent cellular effects from mechanisms independent of the mitochondria, however this has not been examined.

Our results indicating that DNP exerts toxic effects to 4-MO mice contrasts with our previous work demonstrating therapeutic effects in young rats after SCI (Jin et al., 2004; Patel et al., 2009). Besides use of a different species, the most notable methodological discrepancies between this work and previous reports were in dosing paradigms. Specifically, our previous work with DNP performed in rat models of SCI utilized either a single pre-treatment or a single acute dose (Jin et al., 2004; Patel et al., 2009), whereas this study utilized daily doses for 1-week post-injury with a 30-min delay from time of injury to administration. DNP has been administered daily for extended periods of time as a treatment in other animal models of neurodegenerative diseases with improved outcomes found after TBI, Alzheimer’s disease, Parkinson’s disease, Huntington’s disease, as well as promoting overall longevity of life (Caldeira da Silva et al., 2008; De Felice and Ferreira, 2006; Hubbard et al., 2018; Lee et al., 2017; Wu et al., 2017). This led us to believe that multiple doses may exert additional protective effects following SCI, however this may not have been the case for younger 4-MO mice. It remains critical for future studies to evaluate the effects of sustained, compared to acute, mitochondrial uncoupling on recovery from neurotrauma to better elucidate the role of mitochondria on both injury and repair. Further, our data highlights potential metabolic differences with age that change the net effects of DNP, which merits further investigation into the pharmacodynamic interaction between age and bioavailability within the cord.

Finally, it is important to note that mitochondrial function is differentially regulated spatially within the nervous system (Sullivan et al., 2004; Yonutas et al., 2015), temporally following injury (Thawer et al., 2013), with cellular specificity (Fecher et al., 2019; Hubbard et al., 2019; Patel et al., 2009), and potentially in a species-specific manner. Not all cells are affected the same by age which could result in a differential response to DNP in a cell- and aging-dependent interaction. For example, increasing evidence indicates that mitochondrial function regulates macrophage and microglia phenotype (Devanney et al., 2020) and we observed that the macrophage response to SCI increases with age (Zhang et al., 2019). The extent to which age and mitochondrial function of macrophages contributes to the current results requires further investigation. Further, aging alone has been attributed to an increase in mitochondrial-derived ROS that is dependent upon cell type and may differentially be affected by DNP. Even within a species subtle genetic differences can exert large effects on mitochondrial function (Gameiro et al., 2013; Ripoll et al., 2012). What can be concluded from the current study is that the influence of mitochondrial function following SCI also depends on age. Understanding how age effects the spatial, temporal, cellular, and species specificity of mitochondrial functions remains to be elucidated and may be essential for successful translation of mitochondrial-targeting therapeutic strategies.

## 7. Conclusions

In conclusion, increased age at time of SCI shifts the response of mild-mitochondrial uncoupling using repeated dosing of DNP from a net toxicity towards therapeutic efficacy. Mechanisms of how increased age changes cellular responses to uncoupling remains to be determined, however, delineating contributions of mitochondrial membrane potential to SCI injury and repair can effectively be investigated through pharmacological delivery of DNP. Results from the current study reiterate that age at time of SCI can significantly change treatment outcomes (Stewart et al., 2019; Zhang et al., 2019) re-enforcing a need to consider age as a biological variable in studies of neurological disorders.

## Supplementary Material

1
