# Supplementary figures and images for: Mitochondria exert age-divergent effects on recovery from spinal cord injury
Source: Exp Neurol. Author manuscript; Available in PMC 2021 Mar 1. (PMC7870583; doi:10.1016/j.expneurol.2021.113597)

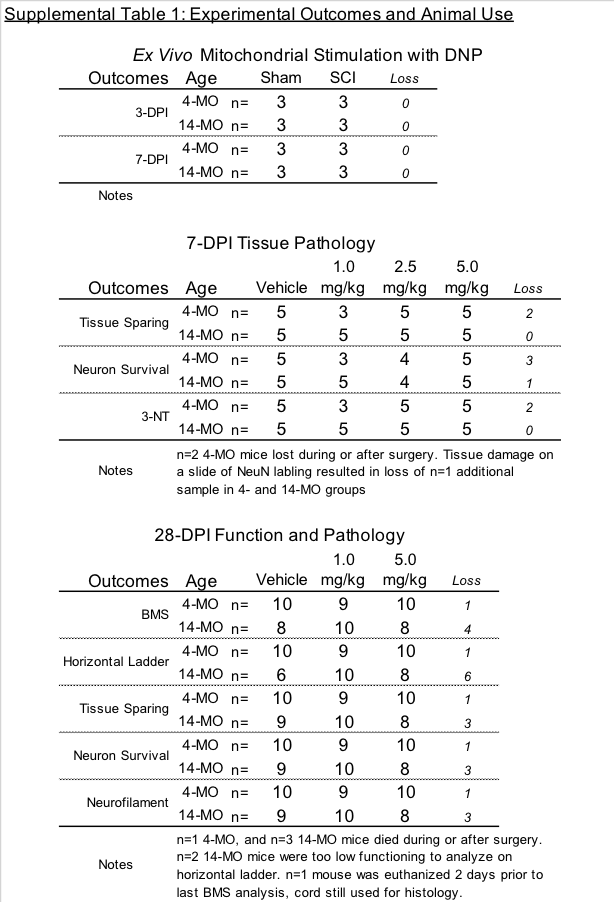

Supplement: 1 [file NIHMS1660903-supplement-1.docx]
